# Still Standing Inside: A Local Idiom Related to Trauma among Namibian Speakers of Khoekhoegowab

**DOI:** 10.3390/ijerph192114323

**Published:** 2022-11-02

**Authors:** Milena Claudius, Elizabeth N. Shino, Sylvanus Job, Daniel Hofmann, Amber Gayle Thalmayer

**Affiliations:** 1 Institute of Psychology, University of Bern, 3012 Bern, Switzerland; 2 Department of Psychology and Professional Counseling, Webster University Geneva, 1293 Bellevue, Switzerland; 3 Department of Psychology and Social Work, Faculty of Health Sciences & Veterinary Medicine, University of Namibia, Windhoek 10026, Namibia; 4 Department of Humanities and Arts, Faculty of Education & Human Sciences, University of Namibia, Windhoek 10026, Namibia; 5 Department of Psychology, University of Zurich, 8050 Zurich, Switzerland; 6 Institute of Psychology, University of Lausanne, 1015 Lausanne, Switzerland

**Keywords:** idioms of distress, trauma, PTSD, Africa, Namibia, cross-cultural psychology, culture, Khoekhoegowab, cultural concepts of distress

## Abstract

Euro-centric psychiatric conceptualizations often ignore the interplay of local with universal factors in psychological suffering. Emic, locally focused perspectives can enrich etic knowledge to provide culturally sensitive care and to better elucidate the role of culture in mental illness. This study explored the idiom *Tsûsa ǃNaeǃkhais xa hâǃnâ/mâǃnâ/ǂgâǃnâhe hâ* (a terrible event has entered a person and remains standing inside), which was understood to relate to experiences of trauma and post-traumatic stress, among speakers of Khoekhoegowab, a southern-African click language. Semi-structured interviews were conducted with 16 participants from six urban and rural communities in Namibia. Questions probed perceptions of the idiom in terms of etiology, course, and risk and resilience factors from a socio-ecological framework. Five key themes were identified using thematic analysis: origin in a shocking event; intrusive recurrence of memories, “it keeps on coming back”; the close interplay between mental and physical suffering; the importance of active engagement in healing through prayer and acceptance; and the role of the community in both alleviating and amplifying distress. Our findings highlight local norms and strategies for adaptive coping, and the benefits of exploring local idioms to elucidate the braiding together of universal and cultural elements in psychological distress.

## 1. Introduction

Understanding the multi-layered experiences of trauma within diverse contexts while refraining from imposing Euro-centric paradigms is a critical step to alleviate suffering and improve appropriate care throughout the world [1,2]. While the discourse on mental health has long been dominated by approaches, samples, and researchers from Europe and the United States, there is growing consensus that psychology research should be internationalized [3,4,5]. Attending to the role of culture as an important dynamic and context-dependent variable can help us to better understand the full complexity of psychological experiences [2], benefiting the study of clinical psychology by elucidating the ways that biology, life experiences, and culture (e.g., local language, religion, values, interpretations) are braided together in the experience of psychological distress and disorders. Slowly but surely, the tradition of applying simple cultural dichotomies (i.e., Eastern vs. Western, individualistic vs. collectivistic) in mental health research is giving way to conceptualizations of culture as dynamic, agentic, and fluid [2], and the integration of etic and emic perspectives is gaining credence for advancing culturally sensitive psychological knowledge and decreasing Euro- and American-centrism. This is especially important given that prognosis for some disorders is worse in wealthier countries [6,7]: the West should not be assumed to have all the answers when it comes to the protection and the recovery of mental health.

### 1.1. Trauma and Posttraumatic Stress across Cultures

Rooted in the Greek language, *trauma* implies a wound or injury. The term is now used frequently for psychological experiences in many contexts. In contemporary psychiatric discourse, it refers to the exposure to actual or threatened death, serious injury or sexual violence [8]. From a biological perspective, psychological trauma affects the nervous system and leads to endocrine changes in the brain and is therefore perceived similar to a physical injury. This view shapes the Western perception of trauma as something that requires healing, while other potential characteristics such as post-traumatic growth or resilience may be neglected [2]. Another long-standing concern is that the Western DSM approach stresses the magnitude of the event, but clinicians observe that individuals can develop trauma-related symptoms after lesser degrees of trauma, which suggests that individual interpretation of an event is more important than objective markers of severity. What constitutes a trauma is shaped by the survivor’s subjective experience [9]. Furthermore, trauma has been described as an indicator of a “plurality of ills” [10] with political and social dimensions, as traumatic stress is also conceptualized as a reaction to various forms of adversity and structural injustices [11,12]. While this expansion of the definition may be important from a social justice perspective, it poses a challenge in defining a clinical construct [10]. Cultural clinical perspectives may allow us to examine to what extent knowledge on traumatic stress is universally applicable and to what degree it needs to be adapted or refuted [2].

Some aspects of trauma appear to be universal [13], yet culture is known to shape its expression and the response to healing and care. Moreover, exposure to the same potentially traumatic experiences does not necessarily lead to the same pathology: Various communities across the globe have shown incredible resilience in the face of violence, tragedy, war, and disaster [14]. In fact, trajectories of resilience might be considered the more normative response in the aftermath of trauma [15]. This means that diagnosis and treatment need to be tailored to the context and that there is great potential to learn from multicultural perspectives. For example, contrary to expectations that higher prevalence of traumatic experiences should be associated with higher rates of post-traumatic stress disorder (PTSD), in South Africa, lifetime prevalence of PTSD was found to be lower than in some European and North American samples where many fewer traumatic experiences are reported [16]. These findings have been replicated in other middle-income countries such as Sri Lanka [17]. Similarly, although Dückers and colleagues [6] found a positive association between exposure to trauma and PTSD prevalence, they identified a ‘vulnerability paradox’ where greater country-level vulnerability in terms of malnutrition, access to sanitation, and income inequality was associated with lower rates of PTSD. The authors proposed that societal context may moderate the pathogenic impact of a stressor: a traumatic event in a high-vulnerability context may be perceived as less shocking than in a country with lower vulnerability [18,19]. This may not be the whole answer, however, as prognosis for schizophrenia has also been seen to have been better in less developed countries [7]. Thus, other explanations for resiliency across contexts should be explored.

The debate as to whether PTSD is an inevitable biological phenomenon [20] versus a Western-created and culture-specific construct [21] is ongoing. While the variability of trauma-related responses across cultures has received growing attention [22], much remains to be understood. There is empirical evidence for the validity of the PTSD construct in some cultures but not in all [23]. Ethnocultural models suggest that the biological response of the nervous system may be universal, whereas affective responses are more culturally mediated [13]. Universally shared biological processes involve the autonomic nervous system with hyperarousal and re-experiencing symptoms [24] or other biomarkers of PTSD, such as neuroendocrine factors, heart rate reactivity [25] or flashbacks [26]. Others, however, question the universality of the construct, arguing that PTSD constitutes a historical product [27] tied to hegemonic scientific, cultural, and moral frameworks [21,28]. Torre and colleagues [29] found that applying classifications of war trauma in post-conflict Uganda could elicit symptoms and worsen suffering. Both universal and culturally specific views of trauma thus offer important perspectives that are considered in cultural-clinical debates.

A Western conception of the psychological response to a traumatic event, including the emergence of PTSD, emphasizes its determinants and treatments at an individual level, leading to the promotion of cognitive-behavioral interventions that are intended to be culture-and context-independent [30]. The current criteria for PTSD in the Diagnostic and Statistical Manual of Mental Disorders (DSM) include exposure to a traumatic event, symptoms of intrusion, avoidance, negative alterations in cognition and mood, and alterations in arousal and reactivity [8]. In the past, avoidance and numbing made up one shared symptom cluster of [31].

Anthropological and cultural clinical perspectives on traumatic stress have shown the advantages and limitations of applying DSM criteria across cultural settings [23,32,33,34]. For example, avoidance, conceptualized primarily as a behavior involving cognitive processes, has been proposed to be a culturally variant response of PTSD [22,35,36,37], which may be overemphasized as pathological in a Western value system [38]. In Nepal, avoiding sites where war atrocities have taken place is considered an adaptive behavior in both healthy and impaired children [9]. Avoidance was, conversely, not observed and not identified as a valid criterion for PTSD among survivors of the South Asian tsunami, who continued to live and collectively recover from the natural disaster in temporary shelters nearby their destroyed homes [39]. Early studies with an indigenous group in Namibia suggest the absence of numbing while certain strategies of avoidance such as temporarily living with family in other villages were culturally acceptable [40]. In the United States, avoidance behaviors have been found to be more common among Latinos in comparison to other racial/ethnic groups [9,41].

Studies in the early 2000s with traumatized West and Central African refugees highlighted intrusive arousal as a distinctive facet in addition to numbing, avoidance, and hypervigilance—suggesting a four-factor PTSD model similar to that found in North American samples [42]. More recently, Fodor and colleagues [43] also reported evidence for the universality of PTSD symptoms among Rwandans. Among Cambodian genocide survivors, recurring, disturbing dreams of the dead induced trauma recall and as such worsened PTSD [44]. Similarly, sleep-related interferences such as nightmares and sleep-paralysis, which triggered fear and catastrophic cognitions, were considered core aspects of a trauma response in other studies with Cambodian survivors [45].

Beyond alterations in arousal, other somatic reactions in response to trauma may have been inadequately considered as a salient aspect of PTSD even though they are frequently reported across cultures [22,46]. For instance, among a sample of children affected by flooding in Namibia, one-third later reported physical symptoms, including stomach aches [47]. Gupta [48] provides an extensive review of somatic symptoms in PTSD. In an effort to combine emic and etic perspectives of trauma, a cross-cultural model of trauma with assessment dimensions partly based on current Western-centric cognitive theories of PTSD (e.g., the emergence of a negative world schema) but also an emphasis on somatic symptoms has been proposed [45]. Among Cambodian refugees, somatic symptoms such as neck soreness, migraine headaches, stomach sensations and dizziness tended to be linked to catastrophizing thoughts and trauma associations [49,50,51].

In Central and Southern Africa high rates of trauma exposure have been reported [16] and meta-analyses have found PTSD to be a major public health problem with prevalence rates up to 22% in sub-Saharan Africa, generally [52]. Given experiences of “mass traumatization” during conflicts in some communities, mental health policies informed by human rights and social justice perspectives are urgently needed [53]. Many authors have pointed to the substantial gaps in reliable knowledge about psychological trauma and its aftermath in Africa, due to a lack of data [52], to the controversy surrounding the conceptualization of PTSD as a universal psychiatric construct [54], and to the difficulty in ensuring multilevel and culturally informed approaches in analysis and interpretations of findings [18,19].

### 1.2. Idioms of Distress

The study of idioms arose as an alternative to the dominant psychiatric discourse and contributes to knowledge of culturally specific views of distress. Idioms of distress [55] are considered a type of Cultural Concepts of Distress which were recently included in the DSM-5 [8] to categorize psychological constructs known to be significantly shaped by culture. Idioms hold cultural meaning, encompass verbal and non-verbal expressions of suffering [56,57] and differ from psychiatric disorders in their pragmatic function and semiotics [57]. Whereas psychiatric disorders are psychometric or operationalized constructs that are intended to be technical and free of personal and social context, idioms have heuristic value in contextualizing the meaning of symptoms [57]. As socially embedded linguistic or nonverbal modes of communicating [55], idioms have both culture-specific [58] and cross-cultural aspects [59,60]. For these reasons, local idioms of distress may be less stigmatizing than Western psychiatric constructs [61] and may be particularly useful for exploring the experience of trauma in diverse contexts. In Nepal, for example, naming local idioms of distress while also offering a Western explanation of PTSD was seen to diminish the unintentional stigmatization of patients, since experiences of trauma are locally associated with bad karma and thus personal culpability [61]. Further exploration of local idioms can contribute to the development of culturally validated screening tools [62], improve communication between patients and medical providers, and support the reduction in stigma for mental disorders.

Research focused on idioms of distress and culturally-rooted explanations of mental illness is still sparse for Sub-Saharan Africa, but some recent findings suggest a notable overlap between local systems of meaning and conventional psychiatric nosology [63]. Among Oshiwambo-speakers in Namibia, for instance, etiological beliefs about *eemwengu* (madness) were identified as having both elements of traditional belief systems and Western medical models of illness. *Eemwengu* has traditionally been interpreted as meaning that someone had been bewitched, cursed, or had a strained relationship with their ancestors. However, more contemporary explanations include having experienced negative, stressful events [63]. In Burundi, Familiar and colleagues [64] found that while the idioms *ihahamuka, ukutiyemera, akabonge* and *kwamana ubwoba burenge* loosely resembled mood and anxiety disorders, participants’ depictions of symptoms differed in quality and quantity from DSM definitions.

A growing body of research also highlights the strong relationship between macro-level factors, structural inequalities, and distress among African-origin individuals [65]. The idiom ‘‘thinking a lot’’ among the Khwe group in South Africa describes thinking about personal and interpersonal problems, yet it also reflects a shared, communal experience of marginalization and social deprivation that includes high unemployment and poverty in comparison to the general population [66]. The authors caution against considering “thinking a lot” as pathological, but instead situate its meaning in the larger socio-economic and political context. Similarly, depressed patients in Zimbabwe attributed the idiom *kunfugisia* (“thinking too much”) to a supernatural cause or social stressors, while somatic symptoms were associated with depression and anxiety [67], indicating both local and contextual meaning as well as universal aspects of this idiom.

With regard to trauma-related idioms, children in Uganda have reported *cen*, which means being haunted by the spirits of those who died during a devastating civil war. Associated with PTSD and severe depression, *cen*’s signs and symptoms include nightmares, sleeplessness, headaches, seeing violent images, and hearing voices [68]. *Cen* was found to express not only individual suffering from extreme poverty, abuse, social deprivation, abandonment, or being forced to kill, but also societal-level suffering and duress [68]. Interestingly, being affected by *cen* thus initiated a communal response to healing in the form of shared rituals. Similarly, in West Africa, *kiyang-yang* was described as a “collective trauma resolution” [69] when, over a 2-year period, about 12% of the population participated in a shared effort to mitigate the psychological effects of political violence. Regarding help-seeking in Burundi, people affected by *guhahamuka* (a set of trauma-related symptoms) reported that visiting a spiritual healer such as a pastor would be most helpful, as would prayer and exposure therapy, but expectations were lower for the potential healing benefits of Western medicine [70].

### 1.3. Namibian and Khoekhoegowab Culture

Little is known about indigenous understandings of psychological disorders generally, or trauma specifically, among Khoisan groups, including Khoekhoegowab speakers in Namibia. Namibia is situated on the Southwest Coast of Africa with a population of about 2.5 million culturally and ethnically diverse people [71] and has endured a long history of colonization, the traumatic oppression of indigenous populations, apartheid policies and a long war for independence [72] which impacted the country’s culture and identity. There are high rates of orphaned children caused by the HIV/AIDS crisis, who are at higher risk for depression [73]. General estimates for depression, anxiety, and schizophrenia with prevalence rates between 6–30% [74] are comparable with findings from other post-conflict settings [75]. Data about psychological trauma is scarce but an ongoing uptick in suicides has raised concerns in Namibia [76]. The suicide rate in sub-Saharan African is thought to be underestimated by the World Health Organization, which relies on generalizations made from small studies [77], but despite this, Namibia is in the top quartile globally [78]. Sexual violence is widespread; reported in a national-representative study by almost 16% of girls and 10% of boys between 13–24 years old [79], and with a lifetime prevalence between 10–27% among women [80,81]. High rates of alcohol abuse [74,82] add to the country’s public health challenges. Namibian mental health care is largely centralized with only two larger psychiatric facilities in the country and a lack of access for most of the rural population [83]. Promising plans have been made to increase services [83], but the general consensus is that the needs for support and treatment far surpass available services [63,74].

While mental health professionals in Namibia are trained in European and American-centered psychiatric frameworks [84], indigenousness belief systems about mental illness shape Namibians perceptions [63]. Moreover, religion and spirituality are highly intertwined with psychology in the Namibian context [85], with many of the 97% of Namibians who identify as Christians [86] also holding traditional belief systems, including beliefs in spirits, ancestral worship, and traditional healing. Some have argued for the inclusion of a spiritual dimension [84] and indigenous belief systems [63] in mental health treatment. Others have pointed towards *ubuntu* or *botho* as an important African philosophy to be considered in mental health practice [87,88], as these encompass the spirituality, interconnectedness, and mutual caring that shape African psychology [88]. An *ubuntu/botho* framework of psychotherapy emphasizes the diversity of human experiences in pluralistic cultures and embraces a holistic, multidisciplinary approach to healing which holds space for both African and Western treatment perspectives [89].

One of the last surviving languages of the Khoe branch of Khoe-Kwadi languages, Khoekhoegowab (also referred to as *Nama, Damara,* or *Haiǁom*) is the second most common mother tongue in Namibia, spoken by about 12% of the population [90]. Speakers of this group retained aspects of hunting and gathering lifestyle into recent times [91]. Genomic studies also show that the Khoisan groups represent some of the most ancient human genetic lineages, indicating that the origin of humankind may be traced back to this part of the continent, which still retains the greatest human genetic diversity on the planet [91,92]. Characterized by four distinct clicks, the language is spoken by two cultural and ethnic groups in Namibia, the Nama and Damara. While the Damara are thought to have been hunter gatherers and pastoralists living in simpler encampments [93], they were displaced with the arrival of the Khoe groups in the area and likely became subjugated by the Nama who tended to have more emphasis on hierarchies and political organization. Nowadays, the economic situation and the lifestyle is comparable among both groups, as members work on farms and in the cities [94].

### 1.4. Goals for the Current Study

The present study builds on prior research with this population [95] and provides a foundation for an ongoing collaborative effort in Namibia to distinguish more universal from more culturally specific elements of mental health. It is the first study we know of to explore a local idiom of distress among Khoekhoegowab speakers, or any Khoisan language speakers. This group provides a strong contrast to typical Western samples in many respects: their historical, social, economic, and geographic context, their language, their local cultural values, which are highly collectivistic in contrast to Western individualism, and their relatively minimal exposure to Western media. While most Khoekhoegowab-speakers in Namibia additionally speak English and/or Afrikaans, we determined during the interviews that Western terms for mental health conditions were largely unfamiliar to participants. A study in this sample thus allows for exploration of more universal versus more locally relevant aspects of mental health.

We report here specifically on how speakers of Khoekhoegowab understand the idiom *Tsûsa ǃNaeǃkhais xa hâǃnâ/mâǃnâ/ǂgâǃnâhe hâ* (“a terrible event has entered a person and remains standing inside”), which was queried within the context of a longer interview investigating local idioms related to three topic areas. We chose to focus on this idiom based on a two-step process. First, broad topics of interest, including post-traumatic stress, were identified as having measurement dissimilarity between Khoekhoegowab speakers and a sample from the United States on a quantitative survey of psychological disorders symptoms [96]. This result led us to wish to better understand this topic area from a local perspective, as it cannot reliably be assessed with imported surveys. As a second step, the third author, a linguist and native speaker of Khoekhoegowab, led extensive discussion of the how distressing events that might relate to ongoing psychological responses are discussed and expressed in the Khoekhoe language. The idiom chosen is, in his professional opinion, the main and perhaps only commonly used way to discuss such experiences. No practical alternatives for this topic were identified. In our analysis, we identify themes from within these spontaneous descriptions in the local language, and also compare them to Western conceptualizations of post-traumatic stress. Thus, we want to learn from emic views to improve etic perspectives, rather than dichotomize the two theoretical lenses. We aim to deeply understand people’s lived experience, and secondarily, to situate local perspectives in a more global context, with reference to the Western construct of PTSD.

## 2. Materials

### 2.1. Sample

Respondents for this study were recruited from six total towns: Windhoek, Karibib, Otavi, and Grootfontein, located in Central and Northern Namibia, which include more Khoekhoegowab speakers identifying as Damara, and Hoachanas and Keetmanshoop in Southern Namibia, which has more Khoekhoegowab speakers identifying as Nama. These sites were chosen in order to access a representative sample of Khoekhoegowab speakers, including an approximately equal number of Damara- and Nama-identified individuals. We also thereby included both larger cities (Windhoek, Keetmanshoop), and smaller towns. In each town, field-based research assistants, schoolteachers who are well known in their local communities, recruited participants. The total sample included 25 interviewees, who were each asked about two of three total topics. Fifteen responded to questions about the idiom discussed here. Interviews took place in private settings arranged by the research assistants, such as in a classroom or office on school grounds after school, in a tent that housed church services during the evening, or on the front porch of a house. Approximately half of the participants identified as Nama, while the other half identified as Damara. Participants’ ages ranged from 21 to 62 years (*M* = 38.13, *SD* = 9.49). Nine participants were male and six were female. Two participants’ highest level of education was grade 7, one of them grade 8, three finished grade 9, two left school after grade 10 and six of the participants completed grade 12. (These grades correspond to the same levels of education as in the United States, e.g., high school is completed with grade 12).

### 2.2. Procedure

Interviews were conducted during the fall of 2020. After providing informed consent, participants engaged in 45–60 min semi-structured interviews with two interviewers. The last author of this report, the principal investigator on the overall study, led the interviews. She was trained in personality and clinical psychology with an emphasis on quantitative methods at a North American university, and has experience conducting semi-structured interviews, leading structured therapeutic groups, and clinical interventions. Due to pandemic-related travel restrictions, she was in Europe and present via WhatsApp video call. The second interviewer, the third author of this report, is a native speaker of Khoekhoegowab who grew up in a village in a Damara-identified region of Western Namibia speaking Khoekhoegowab at home and Afrikaans at school. At the time of the interviews, he was a doctoral student in Khoisan linguistics in Europe, visiting Namibia during an academic break. He was onsite and took the lead in setting the conditions for the interviews, as well as in translating all questions into Khoekhoegowab, and all answers that were given in Khoekhoegowab.

The interview focused on two of three possible topics, alternately between the options, so that similar numbers of participants spoke about each topic. Described in this report are all answers related to *Tsûsa ǃNaeǃkhais xa hâǃnâ/mâǃnâ/ǂgâǃnâhe*. The other two topics were *Kaise ǂgaob ǃnâ tsû hâ /ǂGaoba tsû hâ* (pain in the heart), an idiom thought to be related to depression, and *ǀhorosemâb/s* (drunkard)/ *Ā-ao* (a drinking person, a social drinker), concepts related to alcohol use and abuse. Interviews were audio-recorded with participants’ consent. Participants received a small gift certificate as a token of appreciation for their time. The English content was transcribed verbatim by the first author. Subsequently, transcripts were reviewed by the second interviewer to ensure linguistic accuracy and to ensure the inclusion of Khoekhoe language relevant to the scope of this research.

### 2.3. Interview Guide

To answer our research question, we employed an exploratory qualitative study design. Understanding explanatory models, or culturally determined beliefs about illness and suffering, has been determined to be a critical aspect of culturally sensitive assessment and treatment [97] and many researchers have used explanatory models to explore idioms of distress [98]. In addition, aspects of explanatory models have been incorporated into the DSM-5 cultural formulation interview [99] and included in many clinical cultural capability programs. Therefore, items in the semi-structured interview guide were loosely based on Kleinman’s seminal work on explanatory models of illness [100,101] focusing on participant’s perceptions about the etiology, course, and impact of *Tsûsa ǃNaeǃkhais xa hâǃnâ/mâǃnâ/ǂgâǃnâhe hâ*. Moreover, Bronfenbrenner’s social-ecological framework [102,103] was used to conceptualize the influence of meso-and macro-level contexts on mental health and therefore, inspired questions about stigma or how community members perceived people suffering from *Tsûsa ǃNaeǃkhais xa hâǃnâ/mâǃnâ/ǂgâǃnâhe*.

Specifically, participants were asked if they could think of an example of someone, they had observed to have experienced *Tsûsa ǃNaeǃkhais xa hâǃnâ/mâǃnâ/ǂgâǃnâhe*. The expression was known to all participants. Some participants chose to use themselves as an example, sharing their own experiences, while others described people they knew. Although we sought to understand explanatory models [100] of *Tsûsa ǃNaeǃkhais xa hâǃnâ/mâǃnâ/ǂgâǃnâhe hâ* by asking directed questions, the questions were open-ended, allowing participants to discuss their associations and observations related to the idiom freely.

### 2.4. Qualitative Analysis

Qualitative methods are oriented towards interpreting meaning and experiences [104] and are as such particularly suited for this type of exploration, presuming that reality is a socially constructed (subjective) experience and that a phenomenon must be examined in natural settings without prior hypotheses [105]. Thematic analysis [106,107] may have a unique utility for understanding idioms of distress because unlike other qualitative research methods, it is considered theoretically flexible and can be applied to a range of epistemologies and theoretical orientations [108]. We chose a primarily emic approach to understand perceptions of trauma from the perspective of Khoekhoegowab speakers but as researchers and psychologists trained primarily in Western models of psychology, our analytic lens remained to some degree inherently etic. MAXQDA software [109] served as a data management tool in this analysis.

**Figure A1:**
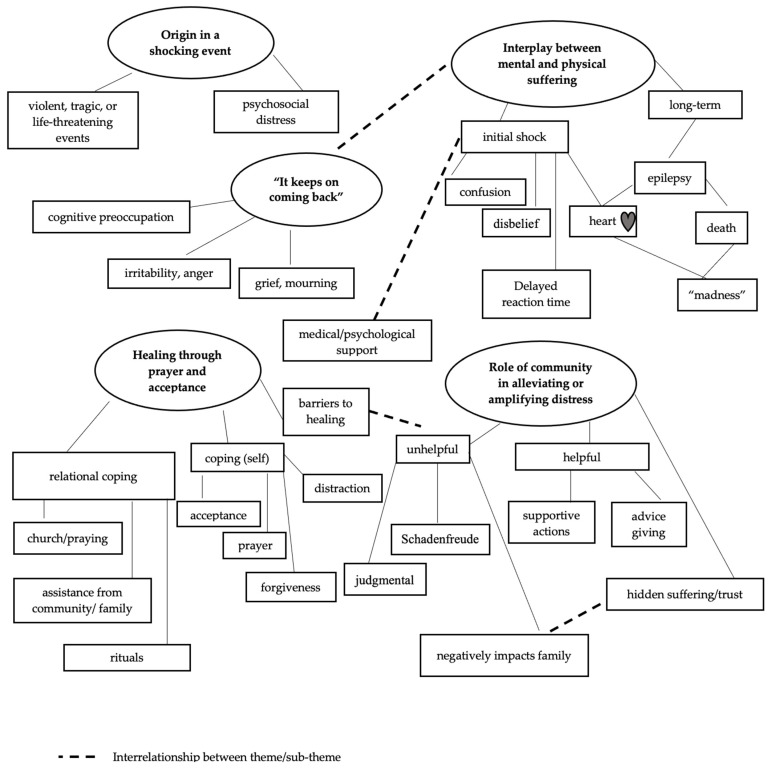
Thematic Map Tsûsa ǃNaeǃkhais hâǃnâ/mâǃnâ/ǂgâǃnâ.

Considering the exploratory nature of the data, an inductive, data-driven approach was chosen whereby codes and themes were identified based on their strong and direct link to the data [106]. In a first step, the first author immersed in the data by reading and re-reading each transcript and taking initial notes about possible codes. In a second step, the data was coded in its entirety which allowed for meaningful organization of the data. An initial codebook was developed. Through discussions among all the authors of the report, only those codes that were relevant to answering the research question were further refined, while others were set aside. Next, codes were collated into potential themes that captured the most salient and meaningful aspects of the data with theme identification occurring at a semantic level. The creation of a thematic map allowed for a visual representation of the data, which was useful to identify relationships between the various themes (see Figure A1 in Appendix A). Then, themes were checked against the data, revised, and reassembled in a way that captured the analytic narrative [106,107]. In addition, throughout the course of this study, team members attended to an important aspect of qualitative research: self-reflexivity–by noting how their socio-cultural positions and subjective lenses and life experiences shaped the interpretation of the data. This started with the interviewers, who made notes about their observations as part of an ongoing reflexive engagement with their positionality and the interview material.

## 3. Results

Our reflexive thematic analytic approach shifted from an initially semantic to a more latent orientation during the analytic process. Multiple points of potential analytic interest were identified through data familiarization. The coding process generated several hundred initial codes that were clustered into patterns of meaning, reducing the codes to a more workable number. The initial codes were clustered into broad patterns. After reviewing the codes, the coded data, and the full dataset, we developed a more theoretical and conceptual analysis which resulted in five distinct themes, which are described further below: (1) Origin in a shocking event, (2) “it keeps on coming back”, (3) the close association between mental and physical suffering, (4) active engagement in healing through prayer and finding acceptance, and (5) the role of the community in alleviating or amplifying distress. For a list of relevant Khoekhoe and Afrikaans terms that came up in depictions of *Tsûsa ǃNaeǃkhais xa hâǃnâ/mâǃnâ/ǂgâǃnâhe*, see Table 1. 

### 3.1. Origin in a Shocking Event

A particularly salient theme for many participants was that *Tsûsa ǃNaeǃkhais xa hâǃnâ/mâǃnâ/ǂgâǃnâhe hâ* originated in a life-changing incident. Such events were often perceived as shocking, unexpected, or tragic and deeply influenced the affected person’s life narrative. Many participants depicted profound experiences of loss, violent in nature, including experiencing the sudden death of a loved one through suicide, homicide, or an accident. For example:

One day…he was found dead, and it was discovered that he had committed suicide by drinking the acid from the car engine….He was the son of a teacher and then the teacher was in school. But then when the teacher returned, they found him dead already and then the mother was a mess, but it was a shocking incident for all of them.

In some cases, participants recalled terrifying experiences such as having survived an attempted kidnapping or being traumatized after having been imprisoned. One participant recalled:

The incident started off when a group of boys … threw sand on the car that was passing by and these boys ran away after doing that and then, the car stopped somewhere in front. And when everybody ran, [I] did not run because [I] did not do anything but then these men who stopped the car and came down thought it was [me] and then grabbed [me] and wanted to force [me] into the trunk of the car. [I] was fighting, like grappling with them and then later, some man, a passerby helped [me] out of that situation. And this is what has remained inside [me].

While most participants found that *Tsûsa ǃNaeǃkhais xa hâǃnâ/mâǃnâ/ǂgâǃnâhe hâ* emerged following a violent, disturbing, or frightening event, as described above, a smaller number of responses associated the idiom with psychosocial stressors, for example in the sense of separation, divorce, family estrangement, or unemployment, as in the following case of the loss of work:

He lost his job at a very crucial time. His wife was pregnant, and his daughter was finishing school, so he was under a lot of pressure. So, you could clearly see this person is under stress. He was like the breadwinner in the family, so there was a lot of pressure on him. Because everyone depended on him. So, when he lost everything that’s when the situation started.

Growing up without one’s caretakers or having had other difficult childhood experiences was also mentioned as a possible reason for *Tsûsa ǃNaeǃkhais xa hâǃnâ/mâǃnâ/ǂgâǃnâhe hâ.*

### 3.2. Intrusive Memories: “It Keeps on Coming Back”

Individuals affected by *Tsûsa ǃNaeǃkhais xa hâǃnâ/mâǃnâ/ǂgâǃnâhe hâ* were described as suffering from intrusive, repetitive reminders of their trauma in the form of memories or flashbacks. In this way, *Tsûsa ǃNaeǃkhais xa hâǃnâ/mâǃnâ/ǂgâǃnâhe hâ* was depicted as persistent and hard to become fully free of. For example:

And this picture that [I] saw there, got stuck into [my] mind. [I] couldn’t get over this for a long time and this thing, it cannot get out of me, it’s stuck with me and whenever [I] would sit, when it was new, [I] would get flashbacks of this…. When [I] wake up, [I] think about this and sometimes [I am] watching television and then [I] see somebody being shot on TV, this picture would come back, come back with [me] and when [I] would sleep, [I] would get these memories of the incident.

Likewise, witnessing another person’s loss or suffering was depicted as evoking memories and activating *Tsûsa ǃNaeǃkhais xa hâǃnâ/mâǃnâ/ǂgâǃnâhe hâ*. For instance:

If a woman loses a child, you cannot really say, really say that she is completely healed inside. You know, she’s maybe not healed because as humans, she may, as a human, she may think about this at some point and if another woman’s child dies, she will also, it will remind her of that time, when her own child has died and that will bring back the memory.

The persistent nature of *Tsûsa ǃNaeǃkhais xa hâǃnâ/mâǃnâ/ǂgâǃnâhe hâ* was evident in other forms of cognitive preoccupation, for example, continually thinking or talking about the experience: “That thing that has happened does not leave the person’s mind. So, this person keeps on talking about the same issue, so you will want to talk about something else, trying to take them away from that topic, but they will keep coming back to that topic, they keep talking about that.”

The tenacity of *Tsûsa ǃNaeǃkhais xa hâǃnâ/mâǃnâ/ǂgâǃnâhe hâ* was also visible through the affected person’s emotional reactions, including anger, fear, and feelings of hypervigilance. For example: “So the person gets aggressive, and you can see that this thing is coming up, they think about this again.” Or, “these people may not do anything [to me] or maybe they are going about their own business, but still seeing people in a group, made [me] afraid.” In contrast to such emotional expressions, other participants described withdrawal, isolation, or loss of interest in response to *Tsûsa ǃNaeǃkhais xa hâǃnâ/mâǃnâ/ǂgâǃnâhe hâ:* “This person becomes reserved, they don’t speak to people, it’s more like isolation.” Reminders of the experience were frequently associated with crying, indicating mourning and grief in some cases, as recalled by one participant: “[I] was thinking about him. And even now, if [I] talk about this or see his pictures, my eyes are filled up with tears, especially when [I] visit the cemetery for another person’s burial.” Interestingly, although clearly implied, the emotion sadness was never directly mentioned. Instead, somatic expressions were often used, as when one participant articulated a deep sense of hurt related to a romantic loss with the words, “[my] heart pains, and it pains [me].”

### 3.3. Close Association between Mental and Physical Suffering

Another theme concerned participants understanding of the ways in which *Tsûsa ǃNaeǃkhais xa hâǃnâ/mâǃnâ/ǂgâǃnâhe hâ* could manifest inside the person. Participants described the progressive nature of the condition, from an initial shock to long-term impacts, in which the psychological experience of *Tsûsa ǃNaeǃkhais xa hâǃnâ/mâǃnâ/ǂgâǃnâhe hâ* could become evident in the body if not relieved. A precursor to such long-term effects was suggested in terms of initial, short-term reactions which signaled being overwhelmed in one’s capacity to cope. As one participant described:

In the beginning, in the first days, it’s very difficult to handle this. It affected [my] work in that [my] reaction time to events around [me] was affected. I found it a little bit, I had a delayed reaction. Maybe if somebody would enter [my] office and … is speaking to [me], [I would] not immediately react or answer. It’s only after a while that [I] realize this person is speaking to me. Maybe [I] will be picking up a phone and then somebody’s on the phone and then the person starts speaking, but then you don’t realize that the person is speaking [to you] …

Fear associated with the event was seen as potentially directly affecting the heart, “immediately after this happened, she got scared so much that she developed heart problems.” Similarly, over a longer period of time, *Tsûsa ǃNaeǃkhais xa hâǃnâ/mâǃnâ/ǂgâǃnâhe hâ* might present in the body as epilepsy, so that the shocking event literally remains inside the person as a lingering explosive. For example, “So after that incident, she developed epilepsy. So she would just be epileptic; she would get these conditions, epileptic conditions.” Moreover, thinking about a shocking event might activate an epileptic episode, “… this thing is still in him because…it appears that when he thinks about this when this thing comes up in him, he gets the epileptic attacks.” In this way, the trauma and suffering became visible to others. Other participants depicted a demise into madness which fostered a social death, then a physical death, as people in the community became reluctant to engage with the affected person:

He could not even eat any longer or take a bath. It got to a level that we had to feed him food and care for him closely. That is what became of him after prison. After the prison, it really became extreme...This can also make a person go mad, especially, if the shocking event is too close to the person…it “touches” [affects] the heart of the person and can even lead to death from a heart attack or cause various chronic illnesses like epilepsy.

### 3.4. Finding Acceptance: Active Engagement in Healing through Prayer

This prominent theme centered around the ways in which individuals affected by a shocking event can successfully cope and recover. Many participants described the process of healing as active, requiring the victim to find inner strength and access their will to accept the painful experience:

Accepting these several shocking events is really not an easy thing but with time, you can take it step-by-step and with time, you will get out of it, but the most important thing is that you will have to ascertain…and recognize[e] the situation in which you are, it’s something like being aware of your situation…part of accepting and then once you are aware that there is a problem, then you accept it and with time, you can become better.

In some cases, forgiving the self and others was also regarded as a way of mitigating distress, with one participant noting: “He wrote a song, a particular song, relating to that incident saying something like ‘Lord forgive me.’ Lord forgive me and have mercy on me.”

Importantly, both coping in relation to others and self-coping strategies were deemed helpful whereby individual and collective prayer were viewed as primary catalysts towards healing and coming to a sense of acceptance or peace. This finding is highlighted in the following comments: “They got him to church, and they prayed for him, so he became fine mentally.” Or:

…if you are to permanently heal a person, even a woman [who has lost her child], the first thing, the thing that you can do is prayer, that you pray for her and that you speak also with her and give her time to talk about this. This will allow the wound inside to heal over time, permanently.

Another interviewee, after describing the agonizing experience of losing his aunt and uncle in a murder-suicide, reflected on having gained empathy and the ability to advise others:

Knowing what happened was a learning curve…and [I] learned from this incident of how people around [my] uncle felt and…that made [me] think, especially when [I] thought about committing suicide…that [I] might have already done something unimaginable by now without even thinking about anyone around. But [I] learned through that…when [I] hear other people having marital issues…[I] also try to advise them with [my] own experience.

Another interviewee emphasized acceptance while pondering existential questions around life and mortality:

One thing that seems to be difficult for human beings is that we seem to, not receive, not accept things that are, that are always there with us, and we know they are there but whenever they happen, they are kind of fresh to us and we are finding it difficult to accept. One of this is death. That death is always around, and you know people are dying. You also know you are going to die. Anybody is likely to die any time, but when death happens, especially with a close relative, people are always struck by [it]. Some of them not even accepting the situation.

In contrast, an inability to accept the experience was perceived as a barrier to recovery, as articulated by one interviewee, “One of the reasons why sudden things can stay with you is if you cannot accept the situation.” Uncertainty around the circumstances around a death was understood as complicating the process of acceptance. One participant stated:

Now with the case of the one who died by gunshot, six years, and eight months. That one did not find closure and they know the person who did it, but they don’t know, the reasons are not given why this shooting took place. The court case is not finalized and this…makes it difficult for them to accept the whole situation and makes this whole situation last with them.

Interestingly, several participants reported that the acute distress following the shocking event was alleviated by medical or psychological interventions, which were depicted as brief but seemingly effective. One person shared, “So [the suicide of her son] had been troubling inside her, that she became mad, but she got treatment at the hospital and that has helped her.” Coping in the form of distraction and avoidance were also mentioned by a few participants.

While no clear pattern was seen regarding the outcome of *Tsûsa ǃNaeǃkhais xa hâǃnâ/mâǃnâ/ǂgâǃnâhe,* it appeared that for the most part, participants continued to live their lives while bearing the consequences of having had experienced a terrible event, noting, for example, “He could [not] perform his work because of his epileptic attacks […] and he said, he only has a month to live [but] interestingly, he is now getting married.” Some participants described the process of healing as continuous and ongoing, lasting a few years to decades in lengths.

### 3.5. The Role of the Community in Alleviating or Amplifying Distress

The community emerged as a significant source of both support and distress for those who experienced *Tsûsa ǃNaeǃkhais xa hâǃnâ/mâǃnâ/ǂgâǃnâhe hâ*. Participants’ reports suggested a marked difference in how individuals more immediately and intimately related to the affected person in comparison to the greater community. The examples were universally described by our participants with great compassion, but many participants described reactions within the community as being judgmental and demoralizing instead of caring and supportive. While we never heard negative judgment expressed toward a sufferer (and this was also true in our interview topics on alcohol abuse and on a local idiom related to depression), participants often indicated that such attitudes were common in the community. We tentatively understand this paradox as indicating that familiarity and intimacy with the sufferer and the experiences they had faced led to more empathic responses. For example: “People said mostly negative things …. They are pointing fingers at him, the people were saying, he was drinking, that’s why he lost his job. All this stuff. That’s what they do.” Another participant said:

You know, as a family, hearing such things hurt you. Some people did make fun of him. Some people would say, yeah, he was cheating people in society. And for this reason, he deserves [this]…this was very painful for me. It was a young person. As a family we had big dreams for him. And for him to end up this way, it was very hard for us.

Others commented on the community’s expectation that someone move on with life after a shocking event: “People generally expect [you] to get over this [the death of her nephew six years ago], over a certain period of time.” In some cases, participants’ accounts suggested *Schadenfreude,* other’s taking joy in someone else’s plight: “There are others who find this funny or are amusing themselves.”

Community responses were instead perceived as helpful when they included pragmatic support or when community members provided advice or motivating words as demonstrated by the following case:

The community members were very helpful for the most part. For example, whenever this person would get lost, they would help with the searches, together with the police. Sometimes the community would also give advice to the family on possible ways they could get along with this man better. They would for instance advise the family to exercise a little more patience when this person finds himself in a peculiar mood, and not to talk with him in an impatient or aggressive way when he’s already in that state.

The latter quote suggests the supportive components of *ubuntu/botho* such as connectedness, affiliation, and belongingness that are related to psychological health. In addition, sharing responsibilities may ease the burden of caretaking by the immediate family. Showing care and love was also described several times as a helpful response:

[I] would advise that this person is loved and is cared for and that you are in a close proximity with this person, both in terms of physical distance, but also in terms of showing them love that you, if they sit, you sit next to them or even if they are there, you show them more that you care for them. That you also assist them with food and so on and that you also keep them, you give them more love. [Interpreter: she is using the word ǀgūse hâ “to be close”] … more close love, showing love and showing that you’re always around them both physically and also emotionally. You could for example sometimes sit next to them, even if you don’t say anything to each other.

Summarizing the significance of communality, one participant was moved to say:

There were people that were really helping in this situation like giving them love, giving them shelter and everything and being there for them. And for them, or them knowing that there is someone that I can go to, there is someone whose house is open for me or there is an open door for me. Yeah, to know that there is someone that cares for me.

These examples highlight the value of community and interconnectedness related to *ubuntu/botho* and the wish to co-exist in harmony.

### 3.6. Other Results

The responses to two final questions were not included in the thematic analysis but are summarized here to give additional context for the themes. At the end of the interviews, participants were asked if they were familiar with the word “trauma” in either English or Afrikaans, since there is no direct translation in Khoekhoegowab. Some participants said that they did not know this word and others who knew the term were queried if they thought it was the same as or different from *Tsûsa ǃNaeǃkhais xa hâǃnâ/mâǃnâ/ǂgâǃnâhe hâ.* Participants had difficulty with this query and so the question was discontinued and not asked of all participants The few answers were inconclusive, for example: “[It’s] similar, [I] cannot really tell, the finer details of what trauma means but [I] think it’s the same thing, because you get traumatized.” Another noted,

Trauma is probably something that grows in the head, grows somewhere in the head. But then, the other one, *having something inside you*, this is something that comes from some event which is unexpected, which is something like getting frightened so much that you get this, that it goes into you, that it gets internally into you.

Overall, these answers suggested unfamiliarity with this technical term, and reminded the authors of the fact that such psychological language was also previously unfamiliar in the West, but has been more integrated into common parlance in recent decades, now perhaps shaping experiences in ways that have many potential outcomes, from possibly reducing stigma to making the Western medical model seem like an established fact, like the role of viruses in certain diseases, although in fact being less concretely established. As almost none of the participants knew the Afrikaans or English word for trauma, we have reason to believe that the Western construct is not well known, and that descriptions related to the local idiom were not influenced by Western conceptions of trauma.

After all topics (two for each participant as described above) had been queried, the closing question was: “What was it like to talk with us today?” This was asked to give participants the opportunity to reflect on the experience and to feel more contained after disclosing content that was often emotionally laden. It was also added because the Namibian team members, as well many research assistants working on a related survey study, had emphasized the cultural incongruence of asking about these topics. Over and over, we had heard, “we don’t talk about these things here.” Given this, the responses were surprisingly positive. Many of the participants expressed gratitude and relief about having had the opportunity to share their stories, while also acknowledging that it was unusual to have done so. Again, associations with the body were drawn, with release being felt in the chest or heart area, in particular. One male participant said, “[I’m] feeling good because it’s like at least something off my chest because I never talked about this to anyone so I’m feeling like it’s a relief and very proud to share this with other people as well.” Another stated, “[I] enjoyed talking with [you]…talking eases the load that you have on the heart, or the load you get with these problems, so if you talk more, it eases it.” Some noted trepidation prior to the interview but having felt at ease during the process. One added blessings as follows:

By answering these questions, [I] also learn things and … many people being interviewed here, learn things about this...And [I’m] appreciating it, and [I’m] wishing [you] well. God led [your] work before, going forward and…people also trusted [you]…[I] wish [you] well and [that] God achieves what [you] plan in the future.

## 4. Discussion

In the current study we explored how Namibian speakers of Khoekhoegowab understand the idiom *Tsûsa ǃNaeǃkhais xa hâǃnâ/mâǃnâ/ǂgâǃnâhe hâ*, (“a terrible event has entered a person and remains standing inside”), and how it relates to Western conceptions of post-traumatic stress. Khoisan groups (including Khoekhoegowab speakers) have been of interest in anthropology, evolutionary psychology, and linguistics, due to their ancient lineage in southern Africa and their retention of aspects of their hunting and gathering lifestyle into recent times [91], but they have been little engaged in terms of their views and experiences with regard to mental health. This group provides a strong contrast to typical Western samples in many regards, especially as participants reported virtually no familiarity with the Western term for trauma. This allows for an exploration of an indigenous, locally relevant idiom related to of post-traumatic stress, as a contrast to the current Western understanding. Aspects that are similar suggest potentially more universal aspects of this kind of experience.

Sixteen adult members of the community were interviewed about their views on the etiology, symptoms, behavior, and community perceptions of an experience of *Tsûsa ǃNaeǃkhais xa hâǃnâ/mâǃnâ/ǂgâǃnâhe hâ*. Five key themes were identified from the examples reported: origin in a shocking event, recurrence of intrusive memories, the close association between mental and physical suffering, healing through prayer and acceptance, and the role of the community in amplifying or alleviating distress. Although we are cognizant of the tension inherent in comparing a local idiom with a Western notion of trauma as a reference point, we consider below the notable similarities and differences between what these themes suggest about the idiom *Tsûsa ǃNaeǃkhais xa hâǃnâ/mâǃnâ/ǂgâǃnâhe hâ* and the DSM view of PTSD [8], and consider their implications.

The experience of *Tsûsa ǃNaeǃkhais xa hâǃnâ/mâǃnâ/ǂgâǃnâhe hâ* was described by Khoekhoegowab-speakers as arising out of a shocking event. This is similar to DSM PTSD, except that the DSM more narrowly defines trauma as coming from an actual or perceived threat to life [8]. While such depictions were indeed mentioned by some interviewees, for example, a woman who came close to being kidnapped, or a young man who was abused in prison, the Khoekhoegowab idiom appears to be broader, additionally including complicated bereavement, in particular, as well as other forms of psychosocial suffering. Unlike the idiom *Kaise ǂgaob ǃnâ tsû hâ /ǂGaoba tsû hâ* (pain in the heart), which was also explored in this sample and which was consistently associated with events considered “sad” in a Western context, such as romantic loss and grief, a majority of participants associated *Tsûsa ǃNaeǃkhais xa hâǃnâ/mâǃnâ/ǂgâǃnâhe hâ* with losing a loved one under unexpected and/or violent circumstances, for example, homicide, suicide, or a fatal accident. This is similar to a nationally representative study from South Africa, which indicated that the unexpected death of a loved one accounted for almost 40% of reported potentially traumatic events [16]. Alternatively, for some participants, the initial shock that led to this condition was associated with life-altering disappointments, such as a relationship separation or job loss. While these experiences suggest a departure from the DSM definition of post-traumatic stress, we note that they may match a lay understanding of “trauma” in the West and many other contexts. Quite possibly, a similar range of experiences might be elicited by open-ended questions about “trauma” with community adults in Western contexts.

Our findings corroborate evidence that contextual factors adversely influence mental health [110] and support indigenous African explanatory models indicating that there is generally an external cause for suffering, and that this suffering can be treated [111]. Calls for a paradigm shift in the conceptualization of trauma as existing on a continuum (ranging between chronic contextual stressors to isolated events) have become more salient in psychiatry, psychology, and anthropology [32,61]. Participants in our study rarely seemed to pathologize the experience of *Tsûsa ǃNaeǃkhais xa hâǃnâ/mâǃnâ/ǂgâǃnâhe hâ.* Only in extreme cases, when a person lost contact with reality, were depictions like “mad” or “crazy” used.

Relatedly, the subjective experience as to whether an event is perceived as traumatic, is also related to culture. For example, Tibetan refugees experienced the destruction of religious symbols as traumatic although they did not actually face a threat to their life [112], and family-related trauma was found to be the best predictor of PTSD severity among North-Korean defectors [113]. Among Khoekhoe speakers, where the individual is seen as interconnected with family and community, family separations clearly pose a threat to *ubuntu/botho*, and may as such be perceived as especially traumatic, with similar effects to the death of a loved one. From a non-Western perspective, the ‘personality of the individual is not separate from the social world but rather an embodiment of the various relationships with others, ancestors, or spirits’ [114]. Life and living are co-dependent, and the death of someone close can directly affect one’s own identity, life, and well-being. Thus, the self is threatened from a psychological perspective and what is perceived as “life-threatening” is, in fact, much broader than the Western understanding which refers to a threat to the physical self. This aligns with research pointing to variability in the role of interpretation of what constitutes “trauma” across cultures [22].

The recurrence of intrusive memories and thoughts and the internal replaying of traumatic events that was described by Khoekhoegowab speakers is also a common feature in both Western depictions of PTSD [115] and in other local idioms [61,111]. Our participants’ descriptions reflected a world view that has been altered by a shocking event. This replicates the findings of Rooyen and Nqweni [35] who discuss memory intrusion as a universal feature of PTSD, affecting core schemas related to a person’s sense of self in the world, and of Rassmussen and colleagues [42] who reported intrusions as important features of PTSD among Central and West-African refugees. Losing someone under violent circumstances has also been linked to highly probable intrusive memories in a systematic review [116] and has been described as potentially more harmful among survivors of traumatic loss compared to those who had experienced physical assault or no trauma at all [117]. Participants’ narratives also suggested the presence of DSM PTSD symptoms such as rumination, fear, and anxiety [8].

The close association between mental and physical suffering for our interviewees indicates the interwoven relationship between these experiences. If left unattended or unintegrated, psychological suffering was seen to lead to detrimental health outcomes or actual death. In *Tsûsa ǃNaeǃkhais xa hâǃnâ/mâǃnâ/ǂgâǃnâhe hâ*, the body is often the medium through which suffering is expressed, for example in the form of convulsions. Convulsive movements have also been reported in the trauma narratives of West African migrants [118] or in *Kiyang-yang* in Guinea Bisseau [69]. Similar to the seizures among Khoekhoegowab speakers that were associated with psychological pain, among an indigenous group in India, the metaphor *dhakka* (shock) describes unexpected aversive events that cause strong emotional reverberations, resembling the “bodily sensation of being shaken” [119]. Another danger perceived by Khoekhoegowab speakers was that the heart can be adversely affected by the shock or its aftermath, leading to heart attacks or chronic heart conditions. This converges with how *haypatensi*, associated with heart pain and hypertension among refugees from Sierra Leone, has been described as embodying the trauma of war and displacement [120]. Similarly, somatic experiences induced by traumatic recall were attributed to heart weakness and various abdominal and gastro-intestinal sensations among Cambodians [45]. Our findings support the salience of somatic expressions of trauma [46,121] and the likelihood of somatization as a universal experience [122].

Potentially more culturally-specific is the primary role of prayer and acceptance in healing. Very minimal mention was made of any targeted interventions, such as mental health or religious counseling, medical treatment, healing rituals or other forms of traditional healing. Instead, successful coping generally meant a combination of internally, cognitively coming to terms with the new situation as the reality, and Christian prayer, individually and together with a larger spiritual community. This suggests a cultural script pertaining to the importance of accepting God’s plan. The adaptive wisdom of this script is indicated by prior research highlighting the important role of religion and spirituality in recovering from difficult life experiences. Faith has been shown to counter the adverse effects of adversity and trauma among African-origin populations [123] and to contribute to positive mental health outcomes [124]. Moreover, seeking comfort in religion has been found to be a major coping strategy for refugees from Sub-Saharan Africa [125] and intrinsic religiosity was associated with lower depression among caregivers of people living with HIV in Namibia [126]. Not surprisingly, religion is seen as a main pillar of African resilience and psychological health [124].

The related focus on acceptance, more than on seeking justice, retribution, or specialized treatment, is also a highly pragmatic response in a context with few alternatives, indicating a sober wisdom about the self-destructive potential of ongoing reaction to a traumatic event, whatever justification there might be for such reaction. Acceptance has previously been described as a key aspect of resilience among cancer survivors in South Africa [127], and is an important element in contemporary Western behavioral-oriented trauma treatments [128].

The role of the community in amplifying or alleviating distress is related, in that healing prayer often involved many community members. Depictions of healing from *Tsûsa ǃNaeǃkhais xa hâǃnâ/mâǃnâ/ǂgâǃnâhe hâ* were characterized by tenets of psychological resilience embedded in traditional African views of interdependence and health and the notion of being whole in connection with a relationship to God, others, and the natural environment [129]. As trauma is understood as a problem that affects the whole person and the whole community [130], healing also has communal aspects, with participants describing turning to collective prayer as a powerful source of solace and healing. This relates to the value of *ubuntu/botho* [87,88] in promoting shared resilience and hope.

In its many forms, social support is considered a protective factor in the aftermath of trauma [131]. Communities can serve as sources of resilience, but negative communal responses can also contribute to vulnerability. To date, most research on social support and stigma in Africa has focused on HIV [131,132]. In our study, we found that the community played an important role in providing instrumental and social support for trauma survivors, and to some degree emotional support. Many of our respondents expressed that showing love, tenderness, and care to those who suffer fosters healing. Similarly, among HIV-orphaned youth in South Africa, those with higher social support reported fewer symptoms of PTSD [131]. In contrast, negative perceptions by community members were perceived as hurtful, and contributed to distress in the affected individual [131], similarly to the descriptions provided by our participants.

In summary, *Tsûsa ǃNaeǃkhais xa hâǃnâ/mâǃnâ/ǂgâǃnâhe hâ* resembled the Western construct of post-traumatic stress in terms of its origin, in terms of the cognitive features of intrusive memories, and in symptoms such as rumination, fear, and anxiety. There were correspondences with literature from the West and beyond in terms of the importance of acceptance of the new reality that the individual finds themselves in. In terms of the origin of the condition, our results echo prior scholars in that we note a complex interrelationship between bereavement and trauma; in a post-traumatic response after violent loss, each reaction may exist alongside, intersect with one another, or one or the other may compound the psychological distress [117,133,134].

More different from Western conceptions were the inextricably interwoven nature of mental and physical suffering, and the emphasis on the community. The experiences were generally described in somatic terms; it was notable that participants rarely used emotion words. While many idioms relating sadness to traumatic events have been described in the literature [61], in Khoekhoegowab, words for the emotion of sadness were not used even in these open-hearted discussions. Word for such emotions are few in Khoekhoegowab, but they do include *ǃoa*, a word for grief and mourning which can also refer to the sadness, and which was never used, despite how relevant it seems to the stories described. Despite this, participants made very clear the pain that they or people close to them had experienced. In the eyes of the European-American interviewer, there seemed to be a contradiction between the assertion, and evidence (when told this was the first time a person had spoken about the event) that people do not talk about these things in this community, and how openly and fluidly they spoke about them with us. The Namibian interviewer later clarified that people often discuss the facts related to these experiences; what was unusual in our interviews was expressions of how hurt they had been by them. However, this was done through somatic and cognitive descriptions, rather than using emotion words.

In terms of the importance of the community, the European-Americans on the team also initially experienced this to be a contradiction. People described a lifestyle and perspective defined by *ubuntu/botho* but were reluctant to show their vulnerability and pain to their community. This was particularly true for people who were perceived as leaders or role-models. We tentatively conclude that the extensive interdependence between members of the community may make people more concerned about burdening each other. Namibia, as a post-war country, is a demanding environment [135], and its residents face many social and economic challenges [136]. At the same time, there is minimal institutionalized support, and the main safety network remains the family and community.

More generally, the concept of *ubuntu/botho* is crucial in understanding Afro-centric mental health, and its components of connectedness, social competency, and consciousness may be fundamental to good mental health. The positive effects of connectedness as a form of social support have been well-established [72,137]. However, the potential costs of valuing community over the individual also need to be acknowledged. A value for consensus may imply pressure to conform and to put the needs of the group ahead of one’s own [138]. For example, *ubuntu/botho* conveys obedience and respect, and could imply negative implications in terms of oppressive conformity, group loyalty, and placing the needs of the group first [138]; for example, a young adult might feel pressured to conform despite developing a different lifestyle or beliefs [89]. *Ubuntu* has also been reported to be perceived as lacking for families caring for a member with mental illness [139].

This relates to the other more unique elements here: Christian faith prayer as the primary means of healing, and the lack of medicalized and other targeted approaches. From the outset, these features gave the impression of a community that has no choice but to be self-reliant, where helping and healing are not the domain of professionals or specialists. Although the first reaction from a Western perspective may be to lament the lack of services, we also noticed the ways this situation can strengthen community bonds and empower community members to care for each other. In Western societies, the word “trauma” has arguably become overused, creating a narrative of damage that requires medicalized care. Perhaps some hard experiences could better be reframed as inevitable challenges on the journey of life. The fact that PTSD is more common in the West may have to do with an expectation that official channels can and should make us whole again but obtaining justice and healing treatments cannot bring back the past. Ultimately, we will also have to rely on close others and ourselves, finding inner sources of resilience. While we appreciate the changing concept of resilience and the criticism regarding its use [140], it is evident that people across the world derive meaning from adversity and acknowledge that terrible experiences can lead to increased emotional strength, interpersonal and personal growth, fuller appreciation of life, and positive personality change [141,142,143].

A great example of supporting community resiliency that avoids over-medicalization and works effectively with minimal resources is the “Grandmother benches” created in Zimbabwe by Dr. Dixon Chibanda [144]. In this project, now extended to several countries by the African Mental Health Research Initiative (https://amari-africa.org/), older women are empowered with training in key skills from cognitive behavioral therapy, such as breaking complex situations into individual problems, and in active listening [144]. Disclosing painful experiences to an unknown person or to someone on the periphery of one’s support network may relieve concerns about burdening others. Based on what we heard from participants in terms of the relief of speaking about these experiences, we believe that such a program would be welcome and useful in Namibia.

### Limitations and Future Directions

As with all exploratory research, there were limitations to this project. First, without the restrictions imposed by the pandemic, we might have begun this study by conducting focus groups to generate a broader list of other possible emic idioms that might resemble trauma. Given that these interviews were part of a larger study investigating other idioms of distress, with more time and a sole focus on *Tsûsa ǃNaeǃkhais xa hâǃnâ/mâǃnâ/ǂgâǃnâhe hâ*, more in-depth descriptions may have been generated, providing a fuller picture. Moreover, our analyses were dependent on what our participants chose to share. While they expressed comfort with the interviewers, it is possible that some content was withheld for various reasons. For example, researcher’s identities clearly influence the dynamics of interviewing. Our primary local interviewer was male, which might have inhibited some women interviewees from discussing abusive heterosexual relationships or sexual trauma. Similarly, the research team members navigated in-and outsider positions. All but the third author are outsiders to Khoekhoe culture, and although we sought to cultivate an emic approach in this work, our training as psychologists is grounded in Western psychology, which impacts our questions and interpretations. Other limitations pertain to the demographics of our participants. Although our sample was evenly split regarding gender, age distribution was skewed with most interviewees middle-or-older aged. Thus, our data primarily refers to the experiences of this age group.

Future research could also illuminate how *Tsûsa ǃNaeǃkhais xa hâǃnâ/mâǃnâ/ǂgâǃnâhe hâ* is distinct from other relevant idioms such as *Kaise ǂgaob ǃnâ tsû hâ /ǂGaoba tsû hâ* (pain in the heart). In this vein, we need more evidence to unravel the etiology, onset, development, and impact of *Tsûsa ǃNaeǃkhais xa hâǃnâ/mâǃnâ/ǂgâǃnâhe hâ*. Moreover, given the different emphasis on somatic and psychological symptoms cross-culturally, it could be interesting to explore more general views of the body among populations that use more somatic expressions.

Based on the positive reactions by interviewees about the opportunity to tell their stories, future research could involve the community more directly, for example querying about which topics are of most current relevance. Moreover, in light of cultural values such as *ubuntu/botho*, community-engaged partnerships or participatory-action research may be a way to include Khoekhoegowab speakers in finding solutions to promote mental health. Initiatives like Grandmother Benches might be facilitated through local churches or other formal and informal networks. Resilience-focused projects promoting positive coping by bringing together survivors to nurture community and reclaim agency by making of story cloths [145] might be another useful model. To broaden our socio-ecological lens, future research could also examine how macro-level factors such as policy, economic inequality, or shifting views on gender relations may influence traumatic experiences.

## 5. Conclusions

As psychologists continue to respond to the call towards a more inclusive science [3,4,5], this study adds to scholarship on mental health on the African continent, combining American, European, and African perspectives on psychology, as well as perspectives shaped by the study of clinical and counseling psychology, culture, personality, and linguistics. While mental health research in Africa tends to focus on various manifestations of psychopathology [16], we explored how Khoekhoe speakers spontaneously express distress, and we highlight their resilience in the face of adverse circumstances. Local idioms provide frames that are less pathologizing and remind us to accept the limits of psychiatric classifications and to attend to the meaning of personal stories, cultural beliefs, and explanatory models. Here, we present qualitative findings from a study on *Tsûsa ǃNaeǃkhais xa hâǃnâ/mâǃnâ/ǂgâǃnâhe hâ*, a local idiom of distress related to the experience of trauma and its aftermath. Tragic and often violent loss as well as life-altering psychosocial changes that threatened relationships and roles were depicted as the external causes for this kind of suffering. If not integrated this psychological pain could become embodied in the form of heart conditions or seizures. However, the psychological wounds associated with hardships were often perceived to be part of the course of life and could often be overcome through individual and collective responses, including prayer, pragmatic support, and reframing to find acceptance. Our interviews thus suggested wisdom and generally adaptive response in a context characterized by many losses and stressful life experiences. The words trauma and PTSD were unknown to participants, suggesting that the inclusion of local idioms and local explanatory models is critical as the Namibian health care system expands. We join other scholars in our conviction that the understanding of trauma and post-traumatic stress should not be confined to the discrete category of PTSD, and that the subjective experience of trauma includes the cultural and psychosocial context [32]. Discussions on mental health in African settings should begin with understanding local needs and should closely involve local researchers, leaders, and community members. We encourage others to collaborate with local scholars and communities to enhance global perspectives on trauma and resilience.

## Figures and Tables

**Table 1:** Khoekhoegowab and Afrikaans Expressions Relevant to Depictions of *Tsûsa ǃNaeǃkhais xa hâǃnâ/mâǃnâ/ǂgâǃnâhe hâ*.

Khoekhoegowab Term	Afrikaans	Literal Translation	Descriptive Translation
xū-i xa hâ ǃnâhe hâ		Something lives or exists in(side) you.	A thing/something occupies you on the inside (mentally); it possesses or overwhelms you (mentally).
ǁaixa ra	aggressief	They become aggressive.	They become aggressive.
ǃkhau kai ra		It makes one go insane/crazy.	It can make someone lose their mind.
“ǀgaru”		The person may start talking incohesively, usually in repeated phrases, sentences or topics.	Talking about things that others would view as unreal, or constantly talking about a particular terrible experience or episode from such an experience (nonstop) even long after the event has occurred.
flou	flou	To become epileptic	To become epileptic.
aanvaar ǁoa	aanvaar	Cannot accept	Unable to accept, (from Afrikaans, aanvaar ‘to accept’)
tsâǀkhā ra	touches	It “touches” (the heart of the person).	It likely affects one’s feelings.
	skok	Shocked	To get shocked
ǃaorosa		Lit. to be feared.	Frightening
ǀgūse hâ		“To be close”	To stay close to (be by the side of) the person
nē xū-i ǀkha ǂkhîba kuru tama			(They) don’t make peace with this thing.
ǀguri keep-basen hâ		Lit. have kept (<ENG, keep) this to him/herself	The person has kept this to themselves.
valuable se mûsen tama		(<ENG, valuable), the person does not see themselves valuable anymore	
give up sī ra		Lit. (<ENG, give up) they go on to give up	*They give up (later)*
verlekersen ra	*verlekker*	(Some people are) finding the sweetness for themselves in this (unfortunate) situation.	Some people are amusing themselves with this (unfortunate) situation.
tsû ra		Some people pain themselves (due to an unfortunate situation).	They sympathize with him in his unfortunate situation.

## Data Availability

The transcripts of the interviews, the list of questions, and the codebook can be found: https://osf.io/mhpvg/ (accessed on 25 October 2022).
